# Influence of the Use of a Collagen Membrane Placed on the Bone Window after Sinus Floor Augmentation—An Experimental Study in Rabbits

**DOI:** 10.3390/dj9110131

**Published:** 2021-11-12

**Authors:** Alessandro Perini, Jose Viña-Almunia, Carmen Carda, José Javier Martín de Llano, Daniele Botticelli, Miguel Peñarrocha-Diago

**Affiliations:** 1 Department of Neurosciences, Division of Dentistry, University of Padua, 35122 Padua, Italy; penarrochamiguel@gmail.com; 2 Department of Stomatology, Faculty of Medicine and Dentistry, University of Valencia, 46010 Valencia, Spain; jose-a.vina@uv.es; 3 Department of Pathology and Health Research Institute of the Hospital Clínico (INCLIVA), Faculty of Medicine and Dentistry, University of Valencia, 46010 Valencia, Spain; carmen.carda@uv.es (C.C.); J.Javier.Martin@uv.es (J.J.M.d.L.); 4 Ciber-BBN, Instituto de Salud Carlos III, 46010 Valencia, Spain; 5 ARDEC Academy, 47923 Rimini, Italy; daniele.botticelli@gmail.com

**Keywords:** animal study, sinus floor elevation, bone healing, osteoconductivity, histology, morphometry, collagen membrane, xenograft, antrostomy

## Abstract

Background: We studied the influence on healing of a resorbable membrane covering the osteotomy site after maxillary sinus grafting, evaluated in different regions of the augmented area. Methods: Maxillary sinus augmentation was performed in 24 New Zealand rabbits. Osteotomy, 4 × 6 mm, were performed bilaterally. A collagenated cortico-cancellous porcine bone was used to fill the elevated region. A collagen membrane was randomly placed over the osteotomy site on one side (MG), and the other side was left uncovered (NMG). The animals were euthanized after 2, 4, and 8 weeks; and histomorphometric analysis was performed in eight different regions. Results: New bone percentages were similar in both groups. There were no statistically significant differences. In MG, the overall percentages were 15.6 ± 7.3%, 22.9 ± 6.1%, and 24.9 ± 12.0% after 2, 4, and 8 weeks, respectively. In NMG, the percentages were 11.2 ± 4.5%, 24.1 ± 5.7%, and 24.5 ± 15.7%, respectively. The proportions of new bone in the various regions after 8 weeks were 31 ± 8.9% and 29.9 ± 9.1% in the bone walls region, 25 ± 10.1% and 32.8 ± 9.1% in the submucosa region, 22.6 ± 21.6% and 10.9 ± 11.5 in the middle region, 17.3 ± 14% and 13.4 ± 9.8% in the close-to-window region, and 21.8 ± 11.6%, 19.1 ± 6.4% in the osteotomy region—for MG and NMG, respectively. Conclusions: In both groups the greatest amounts of bone formation occurred near to the pre-existing bone walls, followed by the sub-mucosa region. The smallest amounts were found in the close-to-window region, followed by the central region. The placement of a collagen membrane to cover the osteotomy site did not influence the amount of new bone formation after sinus grafting.

## 1. Introduction

The posterior edentulous maxilla often presents low bone quality and quantity. Maxillary sinus grafting can lead to an adequate amount of bone for placing dental implants. This procedure, first reported by Tatum in 1977 [1] and modified by Boyne in 1984 [2], is now widely accepted as safe and effective, as cited in various review studies [3,4,5,6,7].

In this technique, a window is produced on the lateral wall of the sinus using a round bur, and the sinus mucosa is elevated.

Over the years, many different materials have been used to graft the elevated sinus, such as autologous bone, bone substitutes alone or mixed with autologous bone, and no material at all [5].

Different approaches have also been used for osteotomy closure, such as resorbable or no resorbable membrane, repositioning of the bone window, and leaving the osteotomy site covered only by the flap [3,8,9].

At the moment, there have only been a few clinical studies comparing bone windows covered with a membrane or left uncovered, showing conflicting results. A systematic review with meta-analysis reported better outcomes with the use of a membrane [3]. However, a more recent systematic review with meta-analysis found no differences in survival rate for osteotomy sites protected by a membrane or left uncovered [9]. The main parameter considered in the studies included in those reviews was implant survival rate, which does not give a description of how healing proceeds in the grafted sinus.

Only a few clinical studies evaluated the grafted area from a histological point of view, again reporting controversial results. Some studies reported higher amounts of newly formed bone in the membrane sites [10,11], and others found no differences between membrane and no-membrane sites [9,12,13,14]. All the studies used trephines of limited dimensions to harvest biopsies [9,10,11,12,13,14]. The specimens were harvested at implant insertion sites or from the window area [10,11,12,13,14], leading to difficulty in comparing the results. The small dimensions of the specimens did not allow them to perform a complete evaluation of the grafted area, and this was considered a limitation by some authors [9,12].

In general, the use of membranes in GBR seems to lead to a high rate of soft tissue complications, such as membrane exposure, soft tissue dehiscence, and acute infection, despite the type of membrane used [15,16]. Some authors also reported that GBR sites without exposed membranes reported 76% more bone formation than sites with membrane exposure [17]. No clinical or pre-clinical experimental studies analyzed the healing of a grafted maxillary sinus with or without the use of a membrane covering the osteotomy site, considering the whole grafted area.

Accurate 3D radiographic evaluation of the anatomy of the maxillary sinus seems mandatory, according to many authors, to avoid complications and to provide data for accurate surgical and prosthetic planning [18,19,20]. Some authors also considered modern 3D radiographic imaging and soft tissue overlapping software useful for diagnostic purposes [21] and for designing an accurate surgical guide for osteotomies [22].

Very little and conflicting evidence is available on the influence of a membrane covering the osteotomy site compared it being left unprotected. Most of the evidence is from clinical studies focusing on implants success rates or on histology of limited specimens. Hence, the aim of the present study was to study the influence of a resorbable membrane covering the osteotomy site after maxillary sinus grafting on the healing, evaluated within all the regions of the augmented area and the osteotomy region.

## 2. Materials and Methods

### 2.1. Ethical Statement

The protocol of the present experiment was presented to and approved by the Ethics Committee of Valencia University, Valencia, Spain (2015/VSC/PEA/00236). The ethical rules included by Council Directive of the European Union (53/2013; 1 February 2013) for animal experiments and those indicated by the Royal Decree 223, 14 March and 13 October 1988 were fully adopted. The ARRIVE guidelines were followed for this report.

### 2.2. Study Design and Experimental Animals

Twenty-four New Zealand rabbits, ~24 weeks of age and 3–4 kg of weight, were selected from the Rabbit Farm San Bernardo (Navarra, Spain). Groups of eight animals each were randomly assigned to three different periods of healing, which were 2, 4, and 8 weeks, respectively.

### 2.3. Randomization and Allocation Concealment

An author (D.B.) involved neither in the selection of the animals nor in the surgery performed electronic randomization at www.randomization.com (accessed on 1 February 2017). After the procedure of sinus floor elevation, the osteotomies were randomly covered with a resorbable collagen membrane or left uncovered. The surgeon (A.P.) received the allocation of the collagen membrane after the completion of the sinus lift.

### 2.4. Sample Size

A sample of 7 animals for each follow-up period, subsequently elevated to 8 to balance possible loss of animals, was evaluated as sufficient to disclose differences between groups. In a study in rabbits [23], there was a 10% difference between groups, and this difference might be considered of clinical relevance. Slightly greater variability (standard deviation ± 6%), power = 0.8, and α = 0.05 were applied for sample calculation.

### 2.5. Biomaterials

Both sinuses were augmented with a collagenated cortico-cancellous porcine bone (OsteoBiol Gen-Os^®^, Tecnoss^®^, Giaveno, Turin, Italy; 250–1000 µm).

The osteotomy “membrane” wounds were covered with an equine collagen membrane (OsteoBiol^®^ Evolution 0.3 mm, Tecnoss^®^, Giaveno, Italy), or no membrane for the “no-membrane” sites.

### 2.6. Clinical Procedures

The anesthesia was performed first with ketamine 22 mg/kg (Ketolar^®^; Pfizer, Madrid, Spain) and xylazine 2.5 mg/kg (Rompun^®^, Bayer, Germany) and then propofol 1.5 mg/kg and isoflurane 2%. In addition, morphine 1% 2.5 mg/kg (Braun, Jaen, Spain) was added during surgery. Articaine 4% with epinephrine 1/100,000 (Ultracain forte^®^, Hoechst GmbH, Frankfurt, Germany) was injected locally.

Trichotomy and asepsis of the experimental region were performed, and a 3 mm long sagittal incision was performed on the nasal dorsum. The soft tissues were elevated to expose the nasal bone.

**Figure 1:**
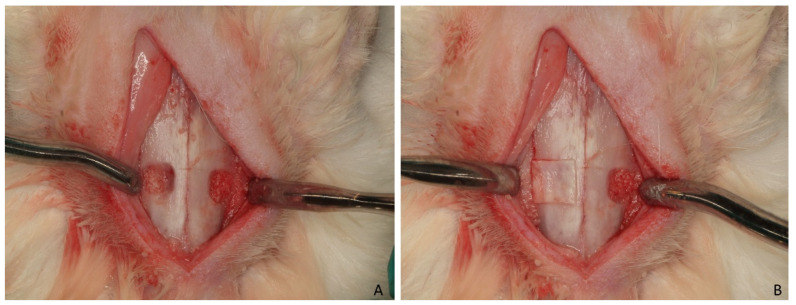
(**A**) Skin and periosteum were elevated separately, and the nasal bone was exposed. The osteotomies were prepared bilaterally, and the elevated spaces were filled with the graft material. (**B**) At one site a collagen membrane was placed to cover the osteotomy, and at the other site the periosteum was directly sutured above the graft material.

Osteotomy sites of 4 × 6 mm, located about 5 mm laterally to the midline and about 10 mm in front of the mesial part of the nasal-frontal suture, were prepared bilaterally in each animal. A round diamond bur was used to prepare the osteotomies and the sinus mucosa was elevated using an elevator of small dimension (Bontempi^®^, San Giovanni in Marignano, RN, Italy). The elevated space was filled with the graft material (Gen-Os^®^; Figure 1A).

At one site a collagen membrane (Evolution—OsteoBiol^®^) was placed to cover the osteotomy (membrane group), and at the other site the periosteum was directly sutured above the graft material (non-membrane group) (Figure 1B).

Vicryl^®^ (Johnson-Johnson, New Brunswick, NJ, USA) was used to close the periosteum, and nylon (Aragó^®^, Barcelona, Spain) was used to close the skin.

Meloxicam 0.2 mg/kg (Normon^®^, Madrid, Spain) once a day for seven days, and buprenorphine hydrochloride 0.02 mg/kg (Buprex^®^, Hull, UK) twice a day for three days, were administered subcutaneously.

### 2.7. Maintenance Care

The animals were kept in individual cages in controlled temperature rooms in the laboratories at the University of Valencia. An accurate check of biological functions and wounds was carried out until the end of the experiment.

### 2.8. Euthanasia

The euthanasia was performed by injecting sodium pentobarbital, 50 mg/kg (Nembutal^®^, Schaumburg, IL, USA).

### 2.9. Preparation of Paraffin Sections 

Samples were fixed in buffered formalin 10% and then decalcified in Osteosoft (Merck KGaA, Darmstadt, Germany). Subsequently, the sample were washed, and the samples were placed in distilled water and then included in paraffin. Sections of about 5 µm thickness were obtained with a microtome (RM2245, Leica Biosystems, Wetzlar, Germany). A central section was stained with scarlet-acid fuchsine and toluidine blue, and then analyzed histomorphometrically.

### 2.10. Histo-Morphometric Analysis

Digital images were taken on a Leica DM4000 B microscope (Leica Microsystems GmbH, Wetzlar, Germany) and merged together.

**Figure 2:**
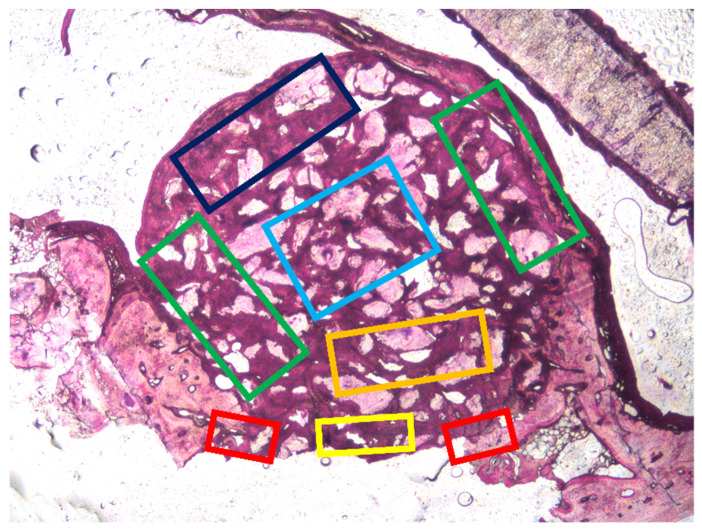
Regions evaluated within the augmented sinuses: (I) close to the bone walls (green rectangle), (II) center of the augmented region (light blue rectangle), (III) subjacent to the sinus mucosa (dark blue rectangle), (IV) and close to the osteotomy region (orange rectangle). Regions evaluated at the osteotomy site: (V) adjacent to the edges (red rectangles) and (VI) the center (yellow rectangle).

An expert assessor (J.M.) took all measurements twice using a lattice with squares of 75 µm superposed on the images. The measurements were performed in the following areas of the elevated space (Figure 2): (i) close to the lateral and medial bone walls (bone walls region), (ii) the center of the elevated area (Middle region), (iii) underneath the sinus mucosa (sub-mucosa region), (iv) and close to the osteotomy (close-to-window region). The osteotomy region was evaluated in 3 different areas: close to the medial and lateral margins and in the central region.

The following tissues were evaluated: newly formed bone, graft material, soft tissues.

### 2.11. Data Analysis

The percentage of new bone formed in the elevated sinus was the primary variable, and that formed in the osteotomy region was the secondary variable. Mean values and standard deviations were calculated for each outcome variable. The IBM SPSS Statistics software, v.19 (IBM Inc., Chicago, IL, USA), was used for applying the Wilcoxon test. The level of significance was set at 5%.

## 3. Results

One animal of the 2-week group showed sinusitis during the healing period and was then excluded from the experiment. No complications were observed during the healing periods in any other animal. The specimen obtained from one animal of the 8-week group had technical problems and could not be used for histologic analysis. The specimens were processed for histological assessment: n = 7 for the 2-week group, n = 8 for the 4-week group, and n = 7 for the 8-week group. Mean values ± standard deviations are used in the text and an asterisk (*) had been added to data when the difference between membrane and non-membrane group was statistically significant.

### 3.1. Grafted Region

**Figure 3:**
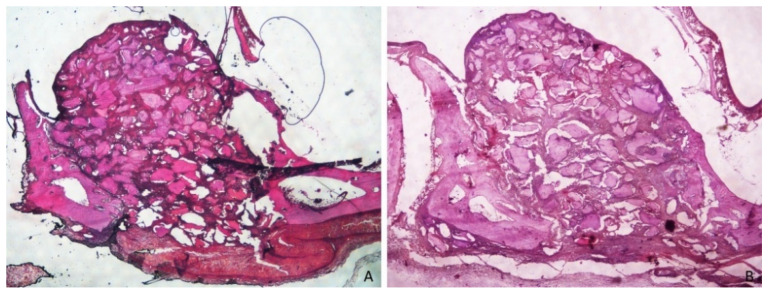
(**A**) Merged images from photomicrographs of sections of decalcified samples representing the healing after 2 weeks in the membrane group. Scarlet-acid fuchsine and toluidine blue. (**B**) Merged images from photomicrographs of sections of decalcified samples representing the healing after 2 weeks the in non-membrane group. Scarlet-acid fuchsine and toluidine blue.

**Figure 4:**
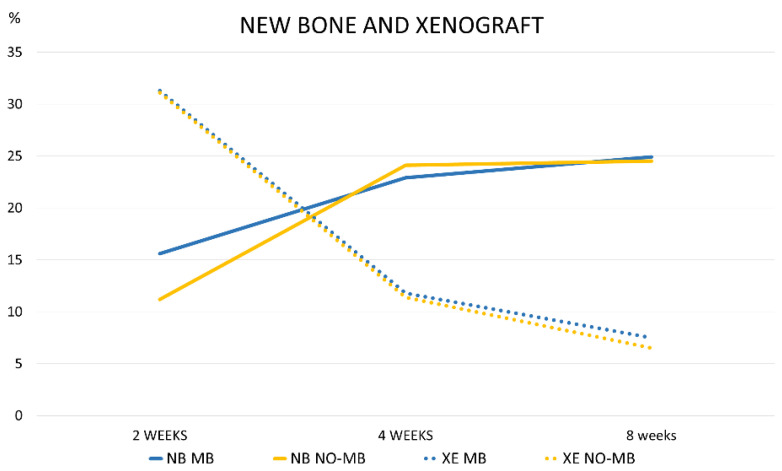
Graph illustrating the percentages of new bone after 2, 4, and 8 weeks of healing in the various regions analyzed within the elevated space. (Abbreviations: NB MB: new bone at membrane site; NB NO-MB: new bone at no-membrane site; XE MB: xenograft at membrane site; XE NO-MB: xenograft at no-membrane site).

After 2 weeks of healing (Figure 3), new bone was present at an overall percentage of 15.6 ± 7.3% in the membrane group, and 11.2 ± 4.5% in the non-membrane group (Figure 4; Table 1).

**Figure 5:**
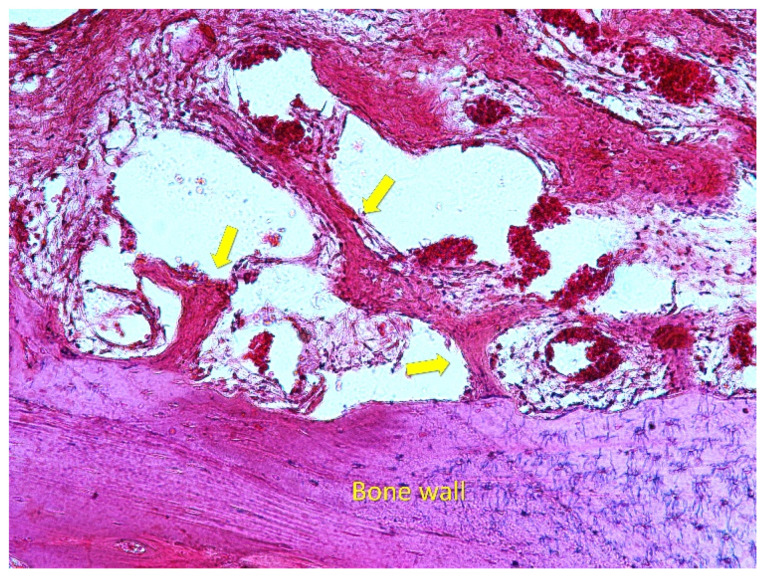
Healing after 2 weeks. New bone was found forming from the sinus walls in both groups. Scarlet-acid fuchsine and toluidine blue. Arrows showing new bone formation.

**Figure 6:**
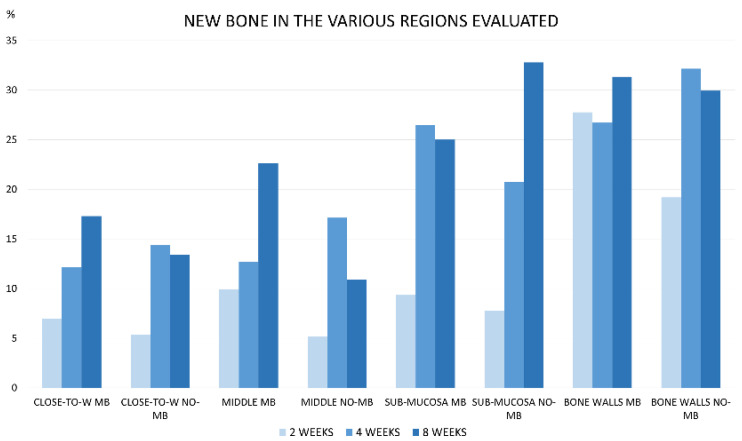
New bone formation in the different regions of the grafted area.

The greater amounts of newly formed bone were detected near the bone walls: 27.8 ± 12% in the membrane group, and 19.2 ± 5.2% in the non-membrane group. The smallest amounts of new bone were found in the no-membrane group in the close-to-window region, with 5.3 ± 4.5%, and in the middle region, with 5.2 ± 4.1% (Figure 5 and Figure 6; Table 2).

The total amount of graft material detected at this time was ~31% in both groups, being less present (~19–20%) near to the bone walls. (Table 1 and Table 2).

**Figure 7:**
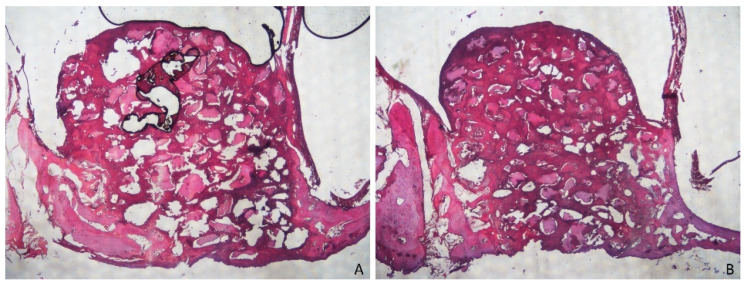
(**A**) Merged images from photomicrographs of sections of decalcified samples representing the healing after 4 weeks in membrane group. Scarlet-acid fuchsine and toluidine blue. (**B**) Merged images from photomicrographs of sections of decalcified samples representing the healing after 4 weeks in non-membrane group. Scarlet-acid fuchsine and toluidine blue.

After 4 weeks of healing (Figure 7), the overall new bone increased to a fraction of 22.9 ± 6.1% in the membrane group, and 24.1 ± 5.7% in the no-membrane group (Figure 4; Table 1).

Again, the higher fractions of new bone were observed in both groups close to the bone walls—a fraction of 27.9 ± 7.6% * in the membrane group, and 32.2 ± 9.5% * in the no-membrane group. The middle and the sub mucosa showed high amounts of new bone—up to 15.3 ± 8.8% and 20 ± 11.9% for the former and 26.4 ± 9% and 21.4 ± 9.6 for the latter, in membrane and no-membrane groups, respectively. Still, the close-to-window area showed the smallest amounts of newly formed bone; however, it increased by ~13% in both groups compared to the previous period of healing (Figure 6; Table 2).

Graft material decreased at this time to ~11% in both groups, and still was less present (~5–7%) near to the bone walls (Table 1 and Table 2).

**Figure 8:**
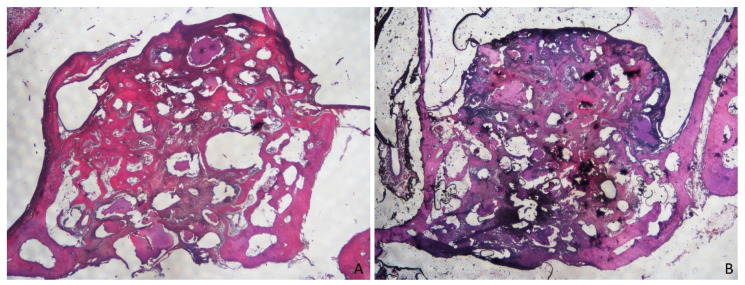
(**A**) Merged images from photomicrographs of sections of decalcified samples representing the healing after 8 weeks in the membrane group. Scarlet-acid fuchsine and toluidine blue. (**B**) Merged images from photomicrographs of sections of decalcified samples representing the healing after 8 weeks in non-membrane group. Scarlet-acid fuchsine and toluidine blue.

After 8 weeks of healing (Figure 8), including all the evaluated areas, new bone slightly increased compared to the 4 weeks period.

The amounts were similar in both groups, being 24.9 ± 12% in the membrane group, and 24.5 ± 4.9% in the no-membrane group (Figure 4; Table 1). The largest amounts of new bone were present in two areas: close to the bone walls at fractions of 31.3 ± 8.9% in the membrane group and 29.9 ± 9.1% in the no-membrane group; and in the sub-mucosal area, with 25 ± 10.1% for the membrane group and 32.8 ± 9.1% for the no-membrane group. The close-to-window and middle regions still showed the smallest amounts of newly formed bone, with percentages of 17.3 ± 14% in the membrane group and 13.4 ± 9.8% in the no-membrane group for the first area, and 22.6 ± 21.6% and 10.9 ± 11.5% in the membrane and no-membrane groups, respectively, in the second one. (Figure 6; Table 2).

Graft material decreased by ~6–7%, and was found to constitute~2–3% near to the bone walls (Table 1 and Table 2).

### 3.2. Osteotomy Region

After 2 weeks of healing (Table 3), newly formed bone was found in three different areas of the window regions, at a rate of 26.4 ± 15.4% at the membrane group, and 18.2 ± 5.9% at the no-membrane group (*p* = 0.063).

The lack of statistical difference was probably due to an animal in the membrane group that showed a little small of newly formed bone and led to a high standard deviation when the whole group was considered. Often, a layer of cortical bone was found partly covering the osteotomy.

After 4 weeks of healing, newly formed bone amounts remained stable for the membrane group (27.4 ± 10.1%), but increased for the no-membrane group to 32.2 ± 10.6%.

After 8 weeks of healing, similar amounts of new bone were found in both groups, showing a slight decrease comparing to the previous healing times, with a rate of 21.8 ± 11.6% in the membrane group and one of 19.1 ± 6.4% in the no-membrane group. In both membrane and no-membrane groups, the osteotomy resulted in residual defects, generally in a central position, involving connective tissue growing through the osteotomy site from the outer regions to the grafted region.

## 4. Discussion

In the present experiment, the only statistically significant difference in bone formation between membrane and no-membrane group was found at 2 weeks in the bone walls region. In all the other regions of the grafted site and in the osteotomy region, no differences were found between the membrane and no-membrane group in any of the periods examined.

The outcomes from the present study disagree with other clinical studies [3,10,11]. A systematic review assessed the treatment’s success at implant installed simultaneously to sinus floor elevation [3]. It was concluded that the best results in implant survival rate were obtained by using a rough surface implant in combination with a membrane. However, this review considered only four studies that compared a resorbable membrane and no membrane, and only the implant survival rate was evaluated [3].

In a clinical study [10], twelve patients received bilateral sinus floor elevation. An e-PTFE membrane was placed to cover one osteotomy site, and the opposite site was left uncovered. At the time of implant installation, the membrane was removed, and biopsies were harvested from the lateral walls of the grafted sites. In the membrane sites, 25.5% of vital bone was assessed, and in the no-membrane sites, 11.9% of vital bone was found. However, bone biopsies were only harvested from the window area and not from the region of the installation.

In another clinical study [11], fifty-one sinus floor elevations were included in the analysis, and resorbable or not-resorbable membranes were used. Additionally, in this study, the biopsies were collected from the osteotomy region after six to ten months post-surgery. Vital bone was found (on average) at 16.9% in the e-PTFE sites, 17.6% at the collagen sites, and 12.1% in the uncovered sites, and no statistically significant differences were found between groups in bone formation or implant survival.

In the present study, after eight weeks of healing, new bone proportion within the whole elevated space was 24.9% in the membrane group, and 24.5% in the no-membrane group. These results are in agreement with a systematic review with meta-analysis [12] that included thirty-seven clinical articles and concluded that the presence of the barrier membrane did not influence the amount of vital bone formation within the grafted sites after sinus floor elevation. However, the authors noted how the location and length of the biopsies can influence the amount of new bone found so that the outcome may not represent the true effect of a barrier membrane. Moreover, several variables might influence the final outcome, and the authors considered as a limitation the harvesting from the alveolar crest used in several of the included studies. In the present study, however, eight different areas were evaluated, and a more comprehensive analysis of the full elevated area was allowed.

In a clinical study [13], only six patients received bilateral sinus floor augmentation using a deproteinized bone mineral from a bovine source. One osteotomy site was covered with a collagen membrane, and the opposite was left uncovered. After eight months, biopsies were collected, with a 2 × 8 mm trephine, from the lateral wall of the grafted sites, without interfering with implants’ placements, and they were histologically evaluated. No statistically significant differences were seen in bone formation between the two groups. Vital bone was 13% and 12% in the membrane and no-membrane groups, respectively.

In another clinical study, [14] a similar experimental design was adopted. The osteotomy sites of nine patients were each covered with a collagen membrane, and those of the other nine patients were left unprotected. After 6 months of healing, biopsies were collected from the lateral walls of the grafted sites. New bone was found at proportions of 30.7% and 28.1% in the membrane and no-membrane groups, respectively. No statistically significant difference was found between the two groups. The heterogeneity of patients regarding the residual bone volume was included in the limitations of the study, along with the depths and locations of the biopsies that might affect the evaluation of the amount of new bone. In the present study, conversely, the histomorphometric assessment was performed in well-defined regions within the elevated space, allowing the evaluation of the areas that were more influenced by the presence of the membrane on the osteotomy site. No statistically significant differences were found after 8 weeks of healing in any of the regions evaluated.

The elevated space was grafted using a porcine collagenated cortico-cancellous bone. A similar biomaterial was also used in other similar experiments in rabbits [24,25,26], and different amounts of new bone were assessed. In one of these studies [24], a membrane was placed subjacent the sinus mucosa in one sinus, and in the opposite sinus, no membrane was used. Both osteotomy sites were protected with a collagen membrane. After 8 weeks of healing, 18.2% of new bone was found in the membrane group, and 26% in the no-membrane group. In another study [25], the bone window was removed after osteotomy preparation and repositioned after sinus grafting at the test sites, whereas the osteotomy at the control site was protected with a collagen membrane. After 8 weeks of healing, 23% of new bone in the repositioned bone window group and 25% in the collagen membrane group were found. In another experimental study in rabbits [26], the osteotomies were prepared for either with drills or with a sonic device. After 8 weeks of healing, new bone was found at 36.3% in the sonic group, and 36.5% in the control group.

In the present study, all osteotomies had residual defects of different dimensions, presenting connective tissue growing from the outer regions through the osteotomy sites to the grafted areas. This is in agreement with the experimental studies discussed above [24,25,26] that reported residual defects at osteotomy sites where collagen membranes were applied. Conversely, in a study in which the bone window was removed and subsequently repositioned in the osteotomy after the grafting procedure [25], very small defects were found, limited to the periphery of the osteotomy site. A longer follow up period and the use of multiple assessment times using standard 3D radiographs or MRI to evaluate bone mineral density could be helpful in evaluating bone formation in this area [26].

In the present study, bone was found in higher proportions near the bone walls of the sinus, and the lowest concentrations of new bone were found in the close-to-window and middle regions. This agrees with several other experimental studies that obtained similar outcomes, and fulfills the rationale that new bone formation comes from pre-existing surrounding bone [25,27,28,29,30,31,32,33]. Newly formed bone at 8 weeks near the bone wall area was found at a fraction of 31.3% in the membrane group and 29.9% in the no-membrane group, whereas in the middle region only had 22.6% and 10.9% new bone, respectively, and the close-to-window region, 17.3% and 13.5%, respectively. The close to bone walls region was also the only region in which a statistical difference was observed between the two groups.

In this study, the xenograft was present after 2 weeks at ~31% in both membrane and no-membrane sites, and progressive degradation was observed during the analyzed healing periods. After 8 weeks, the proportion of biomaterial was ~6–7%. Near to the bone walls, a higher rate of graft resorption occurred as well, with fractions at 8 weeks of 3.5% and 2.1% in the membrane and no-membrane groups, respectively—compared to 10.5% and 8.2% in the middle region and 9.9% and 9.4% in the close-to-window region. This outcome is in agreement with other experiments that used the collagenated cortico-cancellous porcine bone as graft material [24,25], where a reduction in percentages between the various healing periods was observed.

The main limitation of the present study is the phylogenetic distance of rabbits from humans, which limits the applicability of the results to humans [34]. However, the use of animal models allowed accurate histomorphometric evaluations of different areas of the whole augmented sinus. The low number of animals enrolled was due to ethical reasons.

Another limitation of the present study was the small dimensions of the osteotomies. Different types of membranes, and the largest osteotomy region that does not influence the healing without the use of a membrane, should be investigated. Further human studies with larger numbers of patients and experimental studies with larger animals should be performed.

## 5. Conclusions

In both groups, the greatest amount of bone formation occurred near to the pre-existing bone walls, followed by the sub-mucosa region. The lowest amount was found in the close-to-window region, followed by the central region. The placement of a collagen membrane to cover the osteotomy did not influence the amount of new bone formation after sinus grafting.

## Figures and Tables

**Table 1:** Tissue components in percentages for the augmented sinuses in the membrane and no-membrane groups, in regard to the various periods of healing. Mean value ± standard deviation on the first line; median (25 and 75 percentiles) on the second line.

	New Mineralized Bone	Xenograft
2 WEEKS MB	15.6 ± 7.3 18.5 (11.3; 21.2)	31.3 ± 8.1 29.7 (25.6; 36.5)
2 WEEKS NO-MB	11.2 ± 4.5 10.9 (8.8; 12.9)	31.1 ± 3.5 30.9 (28.5; 32.1)
4 WEEKS MB	22.9 ± 6.1 23.0 (17.7; 28.0)	11.8 ± 5.4 10.7 (9.7; 14.0)
4 WEEKS NO-MB	24.1 ± 5.7 24.6 (21.3; 28.8)	11.4 ± 3.1 11.3 (9.8; 12.0)
8 WEEKS MB	24.9 ± 12.0 28.0 (14.9; 33.0)	7.5 ± 3.5 7.7 (6.7; 8.5)
8 WEEKS NO-MB	24.5 ± 4.9 24.7 (23.3; 27.6)	6.5 ± 7.0 3.2 (1.7; 9.6)

None of the differences between membrane and no-membrane groups was statistically significant (*p* < 0.05).

**Table 2:** Mineralized new bone and xenograft percentages (%) in the various regions evaluated after 2, 4, and 8 weeks of healing. Mean value ± standard deviation on the first line; median (25 and 75 percentiles) on the second line.

	2 WEEKS		4 WEEKS		8 WEEKS	
	New Mineralized Bone %	Xenograft	New Mineralize Bone %	Xenograft	New Mineralize Bone %	Xenograft
BONE WALLS MB	27.8 ± 12.0 32.0 (22.1; 35.0)	18.6 ± 8.2 15.9 (14.7; 19.1)	27.9 ± 7.6 * 25.7 (24.9; 29.4)	7.4 ± 5.1 7.2 (3.7; 11.3)	31.3 ± 8.9 35.9 (23.9; 38.7)	3.5 ± 2.2 3.5 (2.3; 5.1)
BONE WALLS NO-MB	19.2 ± 5.2 19.3 (15.7; 22.1)	20.6 ± 11.3 17.1 (13.9; 25.1)	32.9 ± 9.5 * 30.6 (28.2; 38.4)	5.4 ± 3.0 7.0 (4.7; 7.2)	29.9 ± 9.1 32.9 (23.3; 37.4)	2.1 ± 3.1 0.6 (0.0; 3.1)
SUB-MUCOSA MB	9.4 ± 5.7 8.0 (5.7; 12.5)	42.5 ± 6.8 39.8 (37.8; 47.0)	26.4 ± 9.0 27.7 (19.3; 34.9)	13.1 ± 8.4 11.7 (8.5; 15.6)	25.0 ± 10.1 23.3 (19.1; 32.8)	9.5 ± 3.1 10.6 (8.0; 11.7)
SUB-MUCOSA NO-MB	7.8 ± 5.2 5.5 (4.3; 10.9)	36.1 ± 8.9 37.1 (29.3; 42.4)	21.4 ± 9.6 23.9 (12.4; 27.5)	15.1 ± 4.9 14.3 (10.9; 18.4)	32.8 ± 9.1 34.0 (30.8; 38.2)	9.2 ± 8.3 7.9 (2.4; 15.9)
MIDDLE MB	9.9 ± 5.9 12.7 (6.7; 13.1)	32.6 ± 15.8 38.8 (25.4; 42.0)	15.3 ± 8.8 11.2 (8.7; 19.9)	17.5 ± 8.4 16.4 (13.3; 25.3)	22.6 ± 21.6 15.5 (5.0; 36.6)	10.5 ± 5.0 11.9 (7.6; 13.6)
MIDDLE NO-MB	5.2 ± 4.0 5.1(2.5; 8.1)	38.7 ± 10.0 36.1 (32.9; 44.0)	20.0 ± 11.9 18.2 (10.4; 24.1)	17.8 ± 10.6 14.8 (10.4; 20.5)	10.9 ± 11.5 9.2 (2.3; 15.8)	8.2 ± 10.3 6.8 (0.5; 10.3)
CLOSE-TO-WINDOW MB	7.0 ± 5.1 6.3 (3.7; 10.5)	33.7 ± 16.9 36.8 (23.6; 46.0)	13.9 ± 6.3 11.5 (9.6; 17.2)	14.8 ± 8.4 11.6 (9.9; 16.8)	17.3 ± 14.0 21.3 (4.5; 29.2)	9.9 ± 11.0 5.9 (2.0; 14.3)
CLOSE-TO-WINDOW NO-MB	5.3 ± 4.5 6.3 (1.3; 8.6)	42.8 ± 13.7 40.0 (33.1; 50.4)	13.6 ± 8.0 12.2 (7.8; 14.8)	13.1 ± 6.8 13.9 (8.2; 18.0)	13.4 ± 9.8 16.1 (6.6; 18.7)	9.4 ± 13.8 4.9 (0.9; 10.3)

* *p* < 0.05 between membrane and no-membrane groups.

**Table 3:** Mean values ± standard deviations and medians (25%; 75% percentiles) of mineralized new bone percentages (%) in the osteotomy region after 2, 4, and 8 weeks of healing.

2 WEEKS		4 WEEKS		8 WEEKS	
MEMBRANE	NO MEMBRANE	MEMBRANE	NO MEMBRANE	MEMBRANE	NO MEMBRANE
26.4 ± 15.4 27.1 (18.4; 32.2)	18.2 ± 5.9 18.3 (14.9; 21.2)	27.4 ± 10.1 22.6 (20.4; 31.9)	32.2 ± 10.6 31.3 (24.2; 35.7)	21.8 ± 11.6 28.2 (13.8; 30.1)	19.1 ± 6.4 20.1 (16.7; 22.7)

None of the differences between membrane and no-membrane groups was statistically significant (*p* < 0.05).

## Data Availability

The data are available under reasonable request.
